# Acknowledgment to reviewers—January 2024 to December 2024

**DOI:** 10.1093/fsr/owaf004

**Published:** 2025-02-10

The Editors of the *Forensic Sciences Research* (*FSR*) wish to thank the following people, and any other reviewer whose name has been inadvertently omitted, for giving your time and expertise to review papers and facilitate the smooth running of *FSR* between January 2024 to December 2024.

Salem K. Alketbi, *UK*

Abdalla Ahmed Altayeb, *Saudi Arabia*

Muhammad Khan Asif, *Malaysia*

Giovanni Aulino, *Italy*

Ilhan Bahşi, *Turkey*

Valentina Baldini, *Italy*

Pier Matteo Barone, *Italy*

Davide Barreca, *Italy*

Eric J. Bartelink, *USA*

Carolyne Lindsay Bird, *Australia*

Claus Borsting, *Denmark*

Vassiliki A. Boumba, *Greece*

Zuzanna Brozek-Mucha, *Poland*

McCord Bruce, *USA*

Michael Caligiuri, *USA*

Melina Calmon Silva, *USA*

Man Chen, *China*

Xiaohong Chen, *China*

Jillian Conte, *USA*

Cornelius Courts, *Germany*

Shane Darke, *Australia*

Jon M. Davoren, *USA*

Josep De Alcaraz-Fossoul, *Spain*

José Orlando de Almeida Silva, *Brazil*

Júlio de Carvalho Ponce, *Brazil*

Jennifer M. DeBruyn, *USA*

Michele Di Nunzio, *Spain*

Marta Diepenbroek, *Germany*

Danijela Djonic, *Serbia*

Mehmet Dogan, *Turkey*

Chris Douvris, *USA*

Tracey Leigh Dowdeswell, *Canada*

Matias Ignacio Dufek, *Argentina*

Tomasz Dziedzic, *Poland*

Metin I. Eren, *USA*

Recep Fedakar, *Turkey*

Carina Fernandes, *USA*

Ettore Ferrari Júnior, *Brazil*

Neil Fitzgerald, *USA*

Shari Forbes, *Canada*

Lorenzo Franceschetti, *Italy*

Nunzianda Frascione, *UK*

Imanol Garamendi, *Spain*

Daniel Gaudio, *UK*

Brian J. Gestring, *USA*

Marina González, *Brazil*

Cansu Görürgöz, *Turkey*

Ellen McRae Greytak, *USA*

Xueyan Guo, *China*

Yadong Guo, *China*

Mostafa Ibrahim Hassan, *Egypt*

Robert C. Hauhart, *USA*

Guanglin He, *China*

Ning He, *China*

Johannes Hedman, *Sweden*

Samiah Ibrahim, *Canada*

Yangseung Jeong, *USA*

Kurt Jordaens, *Belgium*

Mikoláš Jurda, *Czech Republic*

Efthymia Karantzali, *Greece*

Rajagopal Kavitha, *Malaysia*

Michail Klontzas, *Greece*

Nikolaos Kokkinos, *Greece*

Sunil Kumar, *India*

Marcin Kunicki, *Poland*

Dmitry Kurouski, *USA*

Weibo Liang, *China*

Linchuan Liao, *China*

Feng Liu, *China*

Emanuela Locci, *Italy*

Célia Lopes, *Portugal*

Aurelio Luna, *Spain*

Taís Madeira-Ott, *Brazil*

Naji Arafat Mahat, *Malaysia*

Noridayu Binti Manshor, *Malaysia*

Filipe Peste Martinho, *Portugal*

Anastasia Mitsea, *Greece*

Negahnaz Moghaddam, *Switzerland*

Linton A. Mohammed, *USA*

Muthu Rama Krishnan Mookiah, *UK*

Geoffrey Stewart Morrison, *UK*

Heloise O.M.A. Moura, *Brazil*

Priyanka Nakka, *USA*

Christophe Ndayizeye, *Rwanda*

Efthymia Nikita, *Cyprus*

Bandula Nishshanka, *Sri Lanka*

Zuzana Obertová, *Australia*

Tsukasa Ohuchi, *Japan*

Lucía Ordóñez-Mayán, *Spain*

Tommaso Pacini, *Italy*

Vasanthakumar Packirisamy, *Cayman Islands*

Gurmail S. Paddan, *UK*

Roberto C. Parra, *Democratic Republic of the Congo*

Ana Pego, *USA*

Giulio Pergola, *USA*

Maria Alejandra Perotti, *UK*

Caroline Pollard, *UK*

Nadia Porpiglia, *Italy*

Natalie Reznikov, *Canada*

Olivier Ribaux, *Switzerland*

Tomas Rozsypal, *Czech Republic*

Leticia Rubio, *Spain*

Alberto Salomone, *Italy*

Matteo Scapolan, *USA*

Virginie Scolan, *France*

Nicholas Scurich, *USA*

Francesco Sessa, *Italy*

Giulia Sguazzi, *Italy*

Ravi Kumar Sharma, *India*

H. David Sheets, *USA*

Shaopei Shi, *China*

Jagmahender Singh, *India*

Theresa Stotesbury, *Canada*

Ruiyang Tao, *China*

Duncan Taylor, *Australia*

Richard Thomas, *USA*

Petr Tůma, *Czech Republic*

Ann Van Schepdael, *Belgium*

Kinga Walczak, *Poland*

Meng Wang, *China*

Qi Wang, *China*

Lauren Weidner, *USA*

Hui Yan, *China*

Jun Yao, *China*

Burhan Yarar, *Turkey*

Stefano Zampolli, *Italy*

Hongjian Zhang, *China*

Suhua Zhang, *China*

Vladimir Zivković, *Serbia*

